# A Comparative Study of the Effectiveness of Transdermal Nitroglycerine Patches and Oral Nifedipine in Prolongation of Pregnancy in Women With Preterm Labour

**DOI:** 10.7759/cureus.39106

**Published:** 2023-05-16

**Authors:** Nidhi Goyal, Manjusha Agrawal, Deepika Dewani, Manila Reddy Eleti

**Affiliations:** 1 Department of Obstetrics and Gynaecology, Jawaharlal Nehru Medical College, Datta Meghe Institute of Higher Education and Research, Wardha, IND

**Keywords:** preterm birth, preterm labour, tocolysis, transdermal nitroglycerine patch, nifedipine

## Abstract

Backgrounds

A significant contributor to newborn morbidity and mortality is preterm birth. Several techniques have been employed to identify patients at risk of premature labour. However, these predictors are not always effective because of their multifactorial aetiology. Preterm labour can be suppressed largely through tocolysis. This study compared the effectiveness and safety of transdermal nitroglycerine and oral nifedipine in preventing premature labour.

Methods

This study was done at Acharya Vinoba Bhave Rural Hospital, Sawangi, Wardha, Maharashtra, from December 2020 to November 2022, on 130 women presenting with preterm labour pains between 28 and 37 weeks of gestational age. All the women selected were randomized into two equal groups by using the envelope method. Sixty-five women were given a nitroglycerine patch (Group A), and the rest (65 women) were given an oral nifedipine tablet (group B). The variables studied were mean days of prolongation of pregnancy, treatment outcome, steroid coverage, along with feto-maternal outcomes among both groups.

Results

The percentage of women whose pregnancy was prolonged for at least 48 hours in the nitroglycerine group was 75.3%, and in the nifedipine group it was 93.8%. Failure to achieve tocolysis, defined as delivery within 48 hours, was seen significantly more in the nitroglycerine group (24.6%) than in the nifedipine group (6.1%). The overall foetal outcomes were comparable in both groups.

Conclusion

Oral nifedipine was found to be superior to transdermal nifedipine patches in terms of efficacy and safety in the management of preterm labour, with a better side effect profile.

## Introduction

Preterm labour continues to be a mystery for obstetricians even in the modern era of obstetrics, when there has been a significant advancement in all disciplines, including obstetrical practice. Preterm delivery is defined as delivery less than 37 completed weeks of gestation. It accounts for the majority of neonatal mortality and morbidity [1,2]. Though preterm birth is a global issue, the incidence of preterm birth in developing countries such as India ranges between 10% - 15% of all deliveries, whereas the incidence is about 5% - 10% in developed countries [3].

An established preterm birth is defined as the onset of regular uterine contractions 4 in 20 minutes or 8 in 1 hour, lasting for 40 seconds, after the period of viability and prior to 37 completed weeks of gestation along with cervical changes as per ACOG guidelines [4].

Preterm labour continues to be a mystery for obstetricians even in the modern era of obstetrics, when there has been a significant advancement in all disciplines, including obstetrical practise. Preterm delivery is defined as delivery after less than 37 completed weeks of gestation. It accounts for the majority of neonatal mortality and morbidity [1,2]. Though preterm birth is a global issue, the incidence of preterm birth in developing countries such as India ranges between 10% and 15% of all deliveries, whereas the incidence is about 5% and 10% in developed countries [3].

An established preterm birth is defined as the onset of regular uterine contractions, 4 in 20 minutes or 8 in 1 hour, lasting for 40 seconds, after the period of viability and prior to 37 completed weeks of gestation, along with cervical changes as per ACOG guidelines [4].

A cascade of events leads to the onset of preterm labour by stimulating the myometrial cells. Tocolytic drugs relax the uterus, which delays preterm delivery [5]. The overall goal of tocolytic therapy is to prolong pregnancy by at least 48 hours so that antenatal corticosteroids can be administered to improve foetal maturation and neonatal outcome in the event of preterm birth, delay delivery so that the women can be transferred to a tertiary-care facility where neonatal survival and outcome are improved, and prolong pregnancy itself because the neonatal outcome is directly correlated with gestational age [6-8].

Those in current use include beta-agonists (isoxsuprine), calcium channel blockers (nifedipine), oxytocin receptor antagonists (atosiban), prostaglandin synthetase inhibitors, nitric oxide donors, and magnesium sulphate [9-12].

The most widely used tocolytic medication is nifedipine, which blocks voltage-dependent calcium channels in the plasma membrane, increases calcium efflux from cells, and decreases intracellular free calcium, inhibiting calcium-dependent MLCK (myosin light chain kinase) phosphorylation and myometrial relaxation [13].

Nitroglycerine, also known as glyceryl trinitrate globally, is an alternative recently developed medication that is utilized as a tocolytic (GTN). It is a very volatile, low molecular weight nitrate, also referred to as a nitro vasodilator. Nitroglycerine has a high first-pass metabolism in the liver. A glutathione-dependent organic nitrate reductase in the liver quickly metabolizes the active chemical. Transdermal administration of the drug helps avoid it by ensuring that an adequate amount reaches plasma concentrations [13]. By relaxing the uterine smooth muscles, it defers delivery while also enhancing blood flow to the uterus and placenta.

Some studies present nifedipine as the tocolytic of choice, while few suggest the use of transdermal nitroglycerine due to its convenience of use and fewer side effects [14]. Keeping this background in mind, due to the different mechanisms of action and effects on preterm labour of these drugs, namely nifedipine and nitroglycerine, the present study was aimed at assessing and comparing the effectiveness of nifedipine and transdermal nitroglycerine in the management of preterm labour.

## Materials and methods

Study setting and design

This study was conducted in the Department of Obstetrics and Gynaecology at Acharya Vinobha Bhave Rural Hospital, a tertiary care referral centre located in Sawangi, Nagpur, from December 2020 to November 2022.

Study population and sample size

The formula used to calculate the sample size is n = (Zα + Zβ) 2[P1(1−P1) + P2(1−P2)]/(P1−P2)2, where Zα = level of significance at 5%, Zβ = power of test = 80% = 0.84, P1 = proportion of efficacy of treatment in the transdermal nitroglycerine patch group in the previous study or the pilot study = 85% = 0.85, and P2 = proportion of efficacy of treatment in the oral nifedipine group = 65% = 0.65. The minimum estimated sample size for this study was 63 in each group. A total of 130 women who presented with preterm labour were admitted, and those fulfilling the inclusion criteria were randomized to tocolysis in one of the two groups. Simple randomization was done using the envelope method. Group A: transdermal nitroglycerine patch (n = 65); group B: oral nifedipine (n = 65).

Inclusion criteria

Women presenting with gestational age between 28 and 37 weeks (according to reliable last menstrual period or first-trimester ultrasound) with singleton pregnancy with the cephalic presentation, cervical dilatation between 1 cm and 3 cm, effacement ≥80% with intact membranes, regular painful uterine contractions (minimum of 4 in 20 minutes or 8 in 60 minutes), and no previous tocolytic administration were included in the study.

Exclusion criteria

Women who were excluded from the study were those with documented ruptured membranes, multifetal gestation, women with severe pre-eclampsia, eclampsia, antepartum haemorrhage, polyhydromnios, severe oligohydramnios, severe anaemia, women with systemic diseases like cardiac diseases, diabetes mellitus, renal or hepatic disease and hyperthyroidism, women with foetal complications like congenital anomalies incompatible with life, severe IUGR, intrauterine foetal death, foetal distress, systemic hypotension (blood pressure below 90/60 mmhg), chorioamnionitis, any sensitivity or contraindication to nitrates or nifedipine, or use of any other tocolytics within 24 hours of treatment.

Treatment protocol

Group A

**Figure 1:**
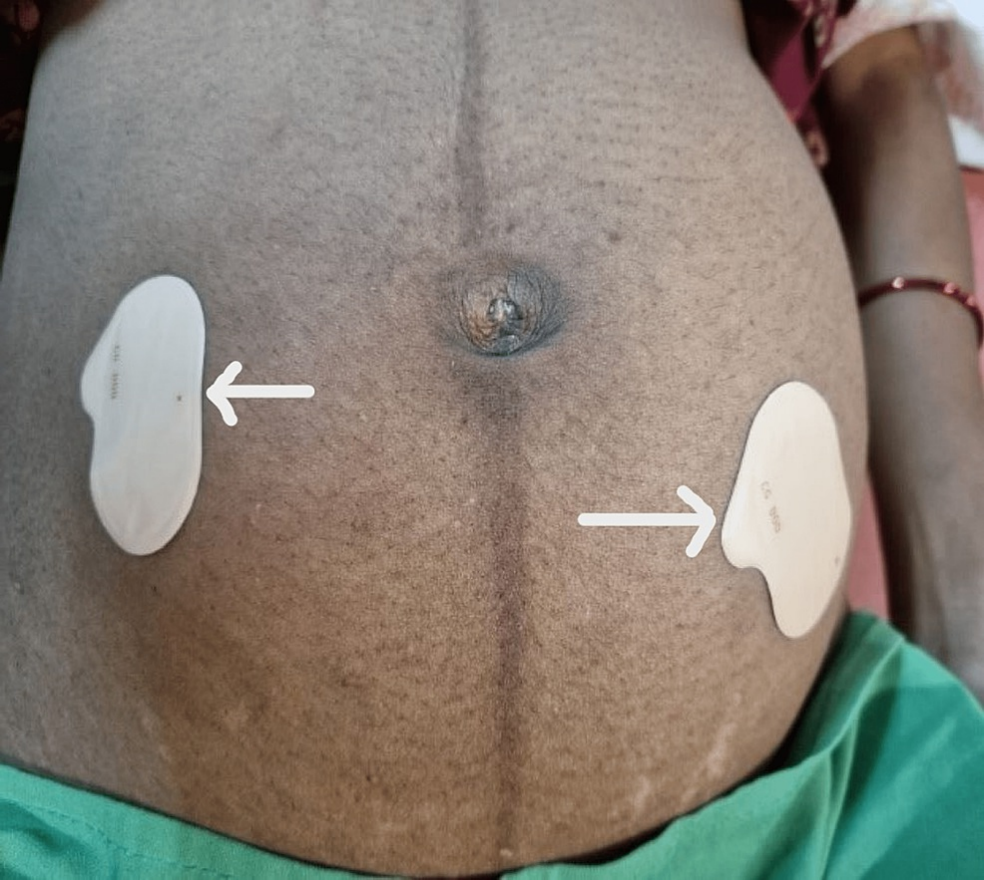
Transdermal nitroglycerine patch (white arrow) applied over anterior abdominal wall.

For subjects allocated to group A, a transdermal nitroglycerine patch (Nitroderm TTS 10) containing 10 mg of nitroglycerine was applied to the anterior abdominal wall (Figure 1). Another 10 mg patch was applied if there was no subsidence in contraction strength and frequency at the end of an hour. A maximum dosage of 20 mg/24 hours was given. Patches were changed every 24 hours until contractions were terminated and for a maximum of seven days.

Group B

Tablet nifedipine (30 mg once stat day, followed by 20 mg after 90 minutes) was given orally. If there was no subsidence in contractions, then a 20 mg tablet of Nifedipine orally every 8th hour for a maximum of seven days was given. To avoid any adverse effects, a maximum dose of 130 mg/day was given.

All patients were started on an injection of dexamethasone 6 mg given every 12 hours intramuscularly for a total of four doses, followed by an injection of ceftriaxone 1 g intravenous 12 hours after admission to avoid infection. Uterine contractions, blood pressure, heart rate, and foetal heart rate were monitored every 15 minutes in the first hour, every two hours until six hours, and then every 12 hours until 48 hours.

Treatment Failure

If the drug could not prolong pregnancy for a minimum period of 48 hours or uterine contractions persisted even after administration of 20 mg of nitroglycerine patch or 50mg of oral nifedipine, then it was classified as a failed treatment.

The study outcomes were recorded in terms of duration of prolongation of pregnancy, mean days of prolongation of pregnancy, steroid coverage, APGAR score, maternal complications, and foetal outcomes.

## Results

The study included 130 women with preterm labour, randomized to groups A: transdermal nitroglycerine patch (n = 65) and group B: oral nifedipine (n = 65).

The baseline characteristics of women in both groups are shown in Table 1. The mean age of women in the nitroglycerine and nifedipine groups was 24.94 ± 3.7 years and 24.4 ± 3.3 years, respectively. There was no significant difference in parity or mean gestational age at presentation. Among various risk factors, a history of preterm labour was seen commonly in women presenting with preterm labour. Both groups had an equal number of women with a history of preterm delivery. The mean gestational age at the time of delivery in the nitroglycerine and nifedipine groups was 34.76 and 35.74 weeks, respectively. The difference among the two groups was statistically significant (p = 0.04).

**Table 1:** Comparability of group A and group B with respect to baseline variables amongst the patients. S: significant (p-value <0.05).

Variable	Group A nitroglycerine (n=65)	Group B nifedipine (n = 65)	p-value
Mean age (years)	24.94 ± 3.7	24.4 ± 3.3	0.18
Primigravida	34 (52.3%)	39 (60.0%)	0.58
Gravida 2	16 (24.6%)	17 (26.1%)	0.58
Gravida ≥ 3	15 (23.0%)	9 (13.8%)	0.58
History of preterm	9 (13.8%)	9 (13.8%)	1.00
Mean gestational age at presentation	33.26 ± 1.9	32.94 ± 1.87	0.51
Mean gestational age at delivery	34.76 ± 2.16	35.47 ± 1.89	0.04, S

The data in Table 2 depicts that the mean days of prolongation of pregnancy were 10.6 days and 17.5 days in the nitroglycerine group and the nifedipine group, respectively, which was statistically significant (p = 0.001). In the nitroglycerine group, 19 (29.4%) pregnancies were prolonged for 48 hours to 7 days, and 11 (16.9%) were prolonged for 7 to 21 days, as compared to 14 (21.5%) pregnancies prolonging for 48 hours to 7 days and 15 (23%) for 7 to 21 days in the nifedipine group. This, however, did not reach statistically significant proportions (p = 0.3). The duration of prolongation of pregnancy exceeding 21 days was observed significantly more (p = 0.01) in the nifedipine group (32/65 women, 49.2%) as compared to the nitroglycerine group (19/65 women, 29.4%).

**Table 2:** Duration of prolongation of pregnancy in two groups S: significant (p-value <0.05)

Duration of prolongation	Nitroglycerine group A	Nifedipine group B	P-value
<48 hours	16 (24.6%)	4 (6.1%)	0.003, S
48 hours -7 days	19 (29.4%)	14 (21.5%)	0.31
7-21 days	11 (16.9%)	15 (23.0%)	0.38
>21 days	19 (29.4%)	32 (49.2%)	0.01, S
Mean days of prolongation	10.67	17.53	0.001, S

Table 3 compares the rate of treatment failure among the two groups. The failure rate in nitroglycerine group A (16/65 women, 24.6%) was significantly higher than that in nifedipine group B (4/65 women, 6.1%) (p = 0.003).

**Table 3:** Treatment outcome among the two groups. x^2^: chi-square.

Treatment outcome	Nitroglycerine group A	Nifedipine group B	x^2^ = 8.50 P = 0.003 significant
Success	49 (75.3%)	61 (93.8%)	
Failure	16 (24.6%)	4 (6.1%)	
Total	65	65	

Table 4 illustrates the maternal side effects reported in both groups. In the nitroglycerine group, headaches were observed in 27 (41.5%) of women, significantly higher than in the nifedipine group (p = 0.006). While maternal tachycardia (pulse > 100 bpm) was observed in 22 (33.8%) women in the nifedipine group B and in 10 (15.3%) women in the nitroglycerine group A, which was statistically significant (p = 0.01). Side effects other than these observed were hypotension, palpitations, dizziness, nausea and vomiting, and a skin rash. The overall incidence of side effects was 60% in the nitroglycerine group and 41.5% in the nifedipine group (p = 0.03).

**Table 4:** Comparison of maternal side effects among the two groups. S: significant (p value <0.05).

Side effects	Nitroglycerine group A	Nifedipine group B	P-value
Headache	27 (41.5%)	7 (10.7%)	0.006, S
Maternal tachycardia	10 (15.3%)	22 (33.8%)	0.01, S
Hypotension	5 (7.6%)	10 (15.3%)	0.17
Skin rash	3 (4.6%)	0 (0%)	0.07
Palpitations	7 (10.7%)	10 (15.3%)	0.43
Dizziness	4 (6.1%)	9 (13.8%)	0.14
Nausea/vomiting	9 (13.8%)	7 (10.7%)	0.59
Overall side effects	39 (60%)	27 (41.5%)	0.03, S

There was no significant difference observed in the neonatal outcomes and complications among both groups, like low birth weight, neonatal jaundice, respiratory distress syndrome (RDS), hypoglycemia, birth asphyxia, sepsis, and neonatal death, as given in Table 5. The APGAR score at one minute was comparable among the nitroglycerine group (7.38 ± 0.92) and the nifedipine group (7.41 ± 0.74). Neonatal jaundice was the most common complication observed in 32/130 (24.6%) of women, followed by respiratory distress syndrome in 18/130 (13.8%) of women.

**Table 5:** Comparison of neonatal outcomes among the two groups. RDS: respiratory distress syndrome.

Neonatal outcome	Nitroglycerine group A	Nifedipine group B	P-value
APGAR score at 1 minute	7.38 ± 0.92	7.41 ± 0.74	0.83
Low birth weight	42 (64.6%)	37 (56.9%)	0.36
Neonatal jaundice	20 (30.7%)	12 (18.4%)	0.10
RDS	12 (18.4%)	6 (9.2%)	0.12
Hypoglycemia	5 (7.6%)	2 (3.0%)	0.24
Birth asphyxia	2 (3.0%)	1 (1.5%)	0.55
Sepsis	8 (12.3%)	6 (9.2%)	0.57
Neonatal death	1 (1.5%)	0 (0%)	0.31

## Discussion

Preterm newborns have high morbidity; some of them are directly related to immaturity due to a lack of pulmonary surfactant causing hyaline membrane disease, while others are indirectly related to immaturity by the excessive use of oxygen leading to retinopathy of prematurity. Evidence suggests that there is a significant reduction in the incidence of respiratory distress syndrome, intraventricular haemorrhage, and neonatal deaths post-administration of a complete corticosteroid course [15]. Therefore, prolongation of pregnancy using an effective tocolytic for at least 48 hours is essential to allow corticosteroid coverage and transfer of the patient to a higher unit.

In the present study, in the nifedipine group, 49.2% of pregnancies were prolonged for more than 21 days, compared to only 29.2% in the nitroglycerine group. The incidence of delivery within 48 hours of initiating treatment was four times more common in group A (transdermal nitroglycerine) compared to group B (oral nifedipine). There was statistically significant more prolongation in the nifedipine group as compared to the nitroglycerine group (p = 0.01). In a study conducted by Dhawle et al. [16], pregnancy in about 56.2% and 48.8% of women in the nitroglycerine group and nifedipine group, respectively, was prolonged for more than 21 days.

The mean prolongation of pregnancy duration was 10.67 and 17.53 days with nitroglycerine and nifedipine, respectively. In 2017, Bala et al. [17] conducted a study where the mean prolongation of days of pregnancy was 13.5 days and 20.74 days in the nitroglycerine and nifedipine groups, respectively. The difference between the two groups was statistically significant. In contrast to the present study, in a study done by Akhtar et al. [18], the mean days of prolongation of pregnancy were higher in the nitroglycerine group (18.45 days) as compared to nifedipine (15.94 days). They explained that these data were statistically significant and implied that nitroglycerine is better than nifedipine.

In the present study, it was observed that the success rate of the drug, which prolongs pregnancy for more than 48 hours, was seen in 49/65 (75.3%) women and 61/65 (93.8%) women in the nitroglycerine and nifedipine groups, respectively. Studies done by Balasubramani and Kamatchi [17] and Dhawle et al. [16] reported that the success rate of nifedipine was significantly higher than that of nitroglycerine in both groups. In the study by Balasubramani and Kamatchi, 72% and 90% of women had successful tocolysis in the nitroglycerine and nifedipine groups, respectively. While in the study by Dhawle et al., successful tocolysis was observed in 68% and 88% of women in the nitroglycerine and nifedipine groups, respectively. The data obtained from the above studies were comparable to the present study.

The most common adverse effect in our study in the nitroglycerine group and the nifedipine group was headache (41.5%) and tachycardia (33.8%), respectively. A study done by Kaur et al. [19] reported that the most common side effect observed in the nitroglycerine group was headache in 42% of women. While the most common side effect in the nifedipine group was tachycardia, observed in 28% of women. In a study conducted by Sharma et al. [20], the most common side effects noted were headache (42.9%) in the nitroglycerine group and tachycardia (45.1%) in the nifedipine group. It was concluded that headaches on nitroglycerine and tachycardia on nifedipine were observed more frequently. Similar results were seen in Salvi et al. [21] and Dhawle et al. [16], where headache was significantly more commonly seen in the nitroglycerine group.

In the present study, 38 (58.4%) neonates from nitroglycerine group A and 23 (35.5%) neonates from nifedipine group B were admitted to the NICU for observation and further management. The most common complication observed in both groups was low birth weight. The observed results were comparable in both groups, with no statistical significance. Studies by Kaur et al. [19], Jamil et al. [22], and Dhawle et al. [16] also reported that low birth weight was the most common neonatal outcome in both groups. There was no statistical significance in neonatal outcomes in either group in any of the above-mentioned studies. The results obtained were in accordance with the present study.

Limitations

The results from the present study need to be further strengthened and validated by multilocational and larger sample-sized research studies to assess optimal dosage, dosage regimes, overall efficacy, and fetal safety. Plenty of risk factors contribute to the causation of preterm labour, some of which remain unknown to the experts. Hence, the measurement of the effectiveness of tocolytics in delaying labour cannot be elucidated by the drug itself.

## Conclusions

Nifedipine is superior to nitroglycerine in terms of prolonging the duration of pregnancy. The mean days of prolongation of pregnancy with nitroglycerine were 10.67 days and 17.53 days with nifedipine. Although the neonatal outcomes were comparable among the two groups, the results obtained from the present study demonstrated that the overall maternal side effects are greater in the nitroglycerine group than in the nifedipine group.
